# Radiation safety issues with high activities of liquid I‐125: Techniques and experience

**DOI:** 10.1120/jacmp.v4i2.2529

**Published:** 2003-03-01

**Authors:** A. F. de Guzman, W. T. Kearns, E. Shaw, S. Tatter, V. Stieber, C. Yates, H. Amadeo, W. H. Hinson

**Affiliations:** ^1^ Department of Radiation Oncology Wake Forest University School of Medicine, Medical Center Boulevard inston‐Salem North Carolina 27157; ^2^ Department of Neurosurgery Wake Forest University School of Medicine, Medical Center Boulevard Winston‐Salem North Carolina 27157; ^3^ Department of Environmental Health and Safety Wake Forest University School of Medicine, Medical Center Boulevard Winston‐Salem North Carolina 27157

**Keywords:** liquid I‐125 source, radiation safety, brachytherapy

## Abstract

The handling of a liquid radioactive source is a procedure that is uncommon for the average clinical medical physicist. A newly approved treatment device utilizes high activities of liquid I‐125 solution as the source of radiation. The radiation safety issues and our experience utilizing high activity liquid I‐125 sources are presented. To date we have treated 22 patients with infused activities ranging up to 26.8 GBq (724 mCi). The careful manipulation of such solutions is important to maintain a safe environment for the patients and the involved medical staff.

PACS number(s): 87.53.‐j, 87.52.‐g

## INTRODUCTION

**Figure 1:**
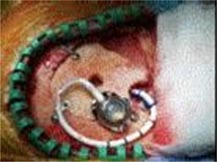
Illustration of GliaSite catheter device placement.

Brachytherapy in resected brain tumors is effective for improving patient survival times. A new device for performing low‐dose‐rate brachytherapy of the margins of resected brain tumors has recently received U.S. FDA marketing clearance.^1^ The treatment technique involves implanting an inflatable balloon catheter (GliaSite® Radiation Therapy System) into the tumor cavity at the time of resection. An infusion port attached to the balloon is secured subcutaneously to the skull and is used to afterload and retrieve fluids from the balloon catheter (see Fig. 1). Brachytherapy is initiated by filling the balloon with up to 26.8 GBq (724 mCi) of Iotrex™, a solution of organically bound I‐125. After completion of the treatment, the Iotrex solution is removed from the GliaSite via the access port.

The use of a liquid radioactive source is unfamiliar to many clinical medical physicists. The radiation safety issues encountered when using a liquid radioactive source are quite different than when using solid, sealed radioactive sources. In addition to the different state of the source, the activity of the source is higher than those normally encountered, up to 27.3 GBq (737 mCi). This paper describes the techniques used when manipulating high activities of Iotrex, the exposure levels for the patient and staff during the procedures, and the general radiation safety concerns treating 22 patients to date.

## METHODS

The GliaSite catheter is placed in the resection cavity during the craniotomy procedure. The balloon is available in three sizes (5, 15, and 35 mL) and is chosen to match the size of the resection cavity. After placement of the GliaSite catheter, the proper activity of Iotrex solution must be ordered. The activity utilized depends on factors such as the balloon size, prescribed dose, treatment depth, and dwell time. We ordered slightly higher activities of Iotrex from the manufacturer than those determined by the nomograms in order to account for residual activity in the vial and in the syringe after injecting the solution into the device.

As is the case when dealing with any source of radiation, film badges, including ring badges, were worn to monitor staff exposure. Shielding, such as leaded L‐blocks and syringe shields were also utilized when handling the radioactive solution. Gloves and other protective garments, such as shoe covers, eye shields, and gowns were worn when manipulating the solution. Personnel from our Environmental Health and Safety (EH&S) department wipe‐tested and opened all incoming Iotrex shipments, as is done with all incoming radioactive material.

The Iotrex was initially delivered in 1 mL unit doses in conical shaped vials containing approximately 7.4 GBq (200 mCi). The physics staff would take the vials to the nuclear medicine department to draw the solution into 5 mL syringe for calibration.^2^ This transfer of the solution from the conical vial to the syringe was performed under a vented hood to reduce the possibility of exposure through volatized I‐125. The physics staff involved with the procedure practiced drawing nonradioactive solution into 5 mL syringes from vials of saline prior to handling the Iotrex solution. This practice increased our familiarity with vials and needles and syringes, which are not the typical tools of your average clinical medical physicist.

The Iotrex solution later became available in preloaded syringes, which eliminated the need for the physicists to transfer the solution from the vial in which the solution was delivered into the 5 mL syringe required for calibration. Once again we ordered slightly higher activities of the Iotrex solution than indicated in the manufacturer's nomograms. This allowed us to account for residual activity left in the syringe after injecting the solution into the device and for residual activity left on the transportation cap of the syringe with which it was delivered.

Once the solution was in a capped, 5 mL syringe it was calibrated by the physics staff using a Capintec CRC‐12 type dose calibrator, (Dial setting 497)^3^ with a NIST traceable calibration which is located in our nuclear medicine department. The calibrated syringe was then transferred in a lead‐lined, wheeled storage container up to the patient's room by a physicist.

Prior to the infusion of the Iotrex the patient is given an oral dose (2 drops of 1 g/mL solution) of potassium iodide (SSKI) to block the uptake of I‐125 by the thyroid should there be a rupture of the balloon or a major spill or leakage of the I‐125 solution. This dosage is continued daily for the course of the treatment. Personnel from our Radiation Safety Department prepare the patient's room by putting absorbent paper on the floor next to the patient's bed and near the doorway of the room. At the time of infusion an additional fenestrated drape is placed over the patients head to allow access to the injection port, but absorb any slight spillage of Iotrex that might occur during the procedure.

The Iotrex solution was injected into the device via the infusion port by either the neurosurgeon or the radiation oncologist. This procedure was practiced several times by the physicians involved with the procedure by using a GliaSite device and nonradioactive saline solution. Physics personnel as well as a member from EH&S were present during the infusion procedure of the Iotrex solution.

After the device is loaded with the radioactive solution, radiation survey measurements are performed around the patient in the patient's room. These are typically performed by our EH&S personnel and are used to determine the amount of time any visitor may stay in the patient's room. A number of items used in the procedure are or may become contaminated with radioactive material and will need to be disposed of properly. These items include, but are not limited to, the syringe used for infusing the Iotrex, the syringe used to add saline to the GliaSite device, gloves, gauze pads, and absorbent pads used to cover the patient during Iotrex infusion.

During the treatment the patient is confined to the hospital room with normal radioactive precautions taken, such as radioactive material warning signs on the patient's room, limited visiting hours, and no removal of bedding, trash or patient clothing from the room without having a radiation survey performed. Personnel from our Environmental Health and Safety department survey the patient room and collect patient urine daily over the course of the treatment.

Patient urine is analyzed throughout the course of treatment, making measurements with a Gamma well counter. It has been estimated by the manufacturer that approximately 0.6% of the administered activity will be excreted via patient urine over the course of a seven‐day treatment due to diffusion of the Iotrex solution through the walls of the GliaSite device.^4^

At the end of the calculated dwell time, the Iotrex is removed from the catheter in a similar fashion as it was loaded. Although the treatment is over, the activity of the I‐125 solution has only decayed by approximately 7% after a typical dwell time of 144 hours (6 days). The precautions used during the infusion of the Iotrex must also be observed during its retrieval from the device.

Once removed, the Iotrex solution and all radioactive waste must be disposed of according to state and/or federal regulations.^5^ At our institution the radioactive waste is stored for a period of at least ten half‐lives until the material can be disposed of with nonradioactive waste. (This may present a problem to smaller institutions which do not have the shielded storage space available to safely contain the waste.)

After a survey of the patient and the room has been performed to be sure that the Iotrex solution has been removed, the device can be removed from the patient's head. This procedure requires a craniotomy to be performed and is done by the neurosurgeons in the operating room. Although the majority of the Iotrex solution has been removed, the device is still radioactive and must be handled accordingly. A member from EH&S or a member of the physics group would retrieve the device from the OR and dispose of it in accordance with state or federal regulations. At our institution, the devices are stored in a shielded area for ten half‐lives until they can be discarded with nonradioactive waste.

## RESULTS

To date, 22 patients have been treated at our institution using the GliaSite Radiation Therapy System utilizing activities from 3.59 to 26.8 GBq (97 to 724 mCi). Dwell times for the patients treated to date have ranged from 98.6 hours to 151.9 hours (4.1 days to 6.3 days). Typical prescription doses are 60 Gy delivered to a depth of 1 cm beyond the balloon surface. Dwell times are determined from nomograms provided by the manufacturer and are based on the balloon size, the prescribed dose, the prescription depth, and the net amount of Iotrex infused into the device.

**Figure 2:**
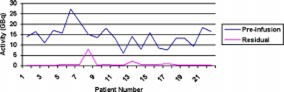
(Color) Measured activity in syringe before and after infusing Iotrex into treatment device.

Residual Iotrex solution is present in the vials in which the solution is delivered and in the syringe after the solution is injected into the treatment device. This residual activity needs to be taken into account when ordering the solution from the manufacturer. The graph (Fig. 2) shows the measured activity of Iotrex in the syringe before and after infusing the solution into the treatment device. The difference between the two values is the net activity infused into the device. The average activity in the syringe prior to injection was 14.32 GBq (387 mCi) with a range from 6.14 GBq (166 mCi) to 27.27 GBq (737 mCi). The average residual activity in the syringe was 851 MBq (23 mCi) with a range of 111 MBq (3 mCi) to 7.96 GBq (215 mCi).

After the device was loaded, members from the EH&S department made measurements of the exposure levels in the room and determined the amount of time that the patient's visitors could spend in the room. Depending on the location of the balloon catheter within the patient's head, the depth of placement, and the total activity infused into the catheter, it was found that one side of the room could receive considerably less exposure than another. This is illustrated in the data from our first five patients in Table I.

**Table I:** Exposure readings at 1 m from patients left and right side after Iotrex infusion.

Activity of Iotrex (GBq)	Exposure reading @ 1 m, right side (μSv/hr)	Exposure reading @ 1 m, left side (μSv/hr)
13.8	28	5
16.2	23	2
10.9	6	2
16.8	40	10
15.1	10	40

As an initial part of our safety program, the medical staff involved with the procedure was subjected to thyroid bioassays and urine analyses to determine any exposure to the Iotrex solution. The tests were performed on the physicists who drew up and calibrated the solution and the physicians who infused the solution into the catheter.

Patient urine was monitored on a daily basis by members of our EH&S department. Although the manufacturer suggested that device leakage may be on the order of 0.1% of the infused activity per day, our analysis of patient urine has consistently fallen below this value with the average total activity excreted being approximately 0.1% for a 6‐day treatment. The measured range has been from 0.05% to 0.16% of the total activity over the entire course of treatment. Typical data from our patient urine analysis is presented in Table II for our first four patients.

**Table II:** Excretion of Iotrex via patient urine.

Activity of Iotrex in patient urine (MBq)	Activity of Iotrex infused into GliaSite device (GBq)						Total percentage of Iotrex in patient urine
	Day1	Day2	Day3	Day4	Day5	Day 6	
13.8	7.4	1.8	3.7	2.6	1.5	1.1	0.13%
16.2	2.2	0.74	2.2	2.2	2.6	1.8	0.07%
10.9	1.8	3.0	1.5	1.8	2.2	0.74	0.10%
16.8	1.1	0.74	3.3	3.0	3.0	1.8	0.08%

Area monitoring using G‐M counters was constantly performed to determine if there was any spillage or radioactive contamination. Such monitoring was performed in the radiation oncology vault where the Iotrex was received, in the nuclear medicine department where the Iotrex is drawn‐up into 5 mL syringes and calibrated, in the patient room where the Iotrex is infused into the treatment device, and on all personnel involved with the procedure. Slight contamination was found on absorbent padding in the nuclear medicine department after our first transfer of Iotrex from a shipment vial to a 5 mL syringe. Since that time there have been no measurable contaminations other than those confined to the absorbent pad covering the injection port.

All 22 patients have successfully completed their prescribed treatments utilizing the GliaSite device loaded with Iotrex solution. There have been no device failures and has been no measurable exposure of the staff during these treatments.

## DISCUSSION

Good communication between the various members from neurosurgery, radiation oncology, radiation protection, nursing personnel, and nuclear medicine is essential to perform safe, efficient treatments using the GliaSite catheter and associated Iotrex solution. Accurate information about the balloon size, fill‐volumes, prescription dose, and prescription depth is needed by the medical physicist in order to obtain the proper activity of Iotrex from the manufacturer. As illustrated in Fig. 2, activities higher than those required for the treatment need to be ordered from the manufacturer due to residual activity left in the shipment vial and the treatment syringe. One patient (#8) had a residual activity in the treatment syringe of 7.96 GBq (215 mCi), due to the fact that the initial treatment device was removed and replaced with a smaller device after the Iotrex had been ordered. Under normal treatment conditions the average residual activity was 518 MBq (14 mCi).

During manipulation of the solution, transferring it from the shipment vial to a syringe or transferring it between the syringe and the treatment device, is when a spill is most likely to occur. For this reason it is important that the members involved with the procedure practice manipulating nonradioactive solutions prior to an actual treatment. In our experience it is better to order the preloaded syringes or have a member from the nuclear medicine department transfer the solution from the shipment vial to the syringe than to have the physicist perform this task. Since we began receiving our Iotrex in preloaded syringes we have not required the assistance of any personnel from the nuclear medicine department. Calibrations are still carried out in the nuclear medicine department using their dose calibrator with a NIST traceable calibration. Careful monitoring of personnel and their surrounding area should be carried out during the various solution manipulations in case of contamination.

Exposure levels in the patient's room ranged from 5 to 50 μSv/hr at a distance of 1 m. As illustrated in Table I, the exposure level will depend not only on the amount of activity in the device but also on the particular side of the patient one is on, due to the various locations of the devices in the patient's heads. By strategically locating the visitor's chairs and with careful instruction from EH&S personnel, visitors were typically able to spend several hours per day visiting with the patient during the course of treatment.

We were initially concerned with personnel exposure to the Iotrex solution not only from a possible spill but also due to any volatility of the solution. For this reason, the medical staff involved with the procedure was subjected to thyroid bioassays and urine analyses to determine any exposure to the Iotrex solution. The tests were performed on the physicists who drew up and calibrated the solution and the physicians who infused the solution into the catheter. After treating several patients and finding no readings above background reading with any of the monitoring techniques utilized, it was decided to discontinue the thyroid bioassays and urine analyses of the staff involved with the procedure.

Constant monitoring of personnel and equipment during the solution manipulations will alert the staff of any contaminations. All materials should be surveyed before being discarded in the regular trash. Contaminated materials such as syringes, gauze pad, and absorbent pads need to be disposed of according to hospital policies and any applicable state and federal regulations.

Monitoring of the activity in the patient's urine was carried out by our EH&S personnel during the course of treatment to detect a rupture of the balloon. As indicated in Table II, there were no balloon ruptures and all measured activity levels were below those predicted by the manufacturer. As is seen in the table, by the activity contained in the patient urine is of no significance to the health care personnel compared to the amount of activity contained in the treatment device.

All patients successfully completed treatment and had the Iotrex solution retrieved from the device without incident. It was decided by the neurosurgeons not to remove the devices from several patients after they had completed treatment. This had no radiation safety implications since all of the patients had exposure readings less than 2 μSv /hr at a distance of 1 m prior to being discharged.

## CONCLUSION

Our experience indicates that high activities of liquid I‐125 solution can be used safely for brachytherapy treatments by observing proper safety precautions and by providing adequate training to the personnel involved with the procedure. Transfer of the Iotrex solution from the shipping vials to a syringe for calibration was safely accomplished with the help and instruction of personnel from our nuclear medicine department. Ordering the Iotrex solution from the manufacturer in preloaded 5 mL syringes which are ready for calibration is advantageous as it requires less manipulation of the solution. Fluid transfers between the syringe and the GliaSite device were safely and easily accomplished via the injection port.

Exposure levels in patient rooms were low enough to allow visitors for several hours per day during the course of treatment. Exposure of staff members involved with the procedure is low as indicated by urine analysis and thyroid bioassays performed during our initial experience and by ring‐badges and other film‐badges worn by the staff. The treatment device performed as expected, with no excessive leakage of Iotrex and no device ruptures as indicated by patient urine analysis.

## References

[1] S. B. Tatter , E. G. Shaw , A. F. de Guzman , M. L. Rosenblum , T. Mikkelson , A. Olivi , and S. Grossman , “Brachytherapy in re‐resected malignant glioma cavities using the gliasite radiotherapy system—A phase I safety and device performance study,” in Proceedings of the American Society of Clinical Oncology, (Lippincott, Williams, and Wilkins, Baltimore, MD, 2000), Vol. 19, p. 160a.

[2] M. T. Gillin , “Calibration of a liquid I‐125 source in a syringe,” J. Appl. Clin. Med. Phys. 3, 218–220 (2002). 12132943 10.1120/jacmp.v3i3.2565 PMC5724600

[3] “The assay of Iotrex™ (I‐125 Radiotherapy Solution),” Technical Information Bulletin No. 1, Doc. No. B30‐100‐012, Proxima Therapeutics, Inc; Alpharetta, GA.

[4] “Biodistribution and dosimetry of Iotrex™ (I‐125 Radiotherapy Solution),” Technical Information Bulletin No. 2, Proxima Therapeutics, Inc; Alpharetta, GA.

[5] North Carolina Regulations for Protection Against Radiation ; The North Carolina Radiation Protection Commission; 15A NCAC 11; North Carolina Department of Environment, Health, and Natural Resources—Division of Radiation Protection; Section 0416 (1993).

